# A machine learning approach to predict intravenous immunoglobulin resistance in Kawasaki disease patients: A study based on a Southeast China population

**DOI:** 10.1371/journal.pone.0237321

**Published:** 2020-08-27

**Authors:** Tengyang Wang, Guanghua Liu, Hongye Lin

**Affiliations:** 1 Department of pediatrics, Fujian Provincial Maternity and Children’s Hospital, Fuzhou, Fujian, China; 2 StarCore Co. Ltd., Fuzhou, Fujian, China; Baylor Scott and White, Texas A&M College of Medicine, UNITED STATES

## Abstract

Kawasaki disease is the leading cause of pediatric acquired heart disease. Coronary artery abnormalities are the main complication of Kawasaki disease. Kawasaki disease patients with intravenous immunoglobulin resistance are at a greater risk of developing coronary artery abnormalities. Several scoring models have been established to predict resistance to intravenous immunoglobulin, but clinicians usually do not apply those models in patients because of their poor performance. To find a better model, we retrospectively collected data including 753 observations and 82 variables. A total of 644 observations were included in the analysis, and 124 of the patients observed were intravenous immunoglobulin resistant (19.25%). We considered 7 different linear and nonlinear machine learning algorithms, including logistic regression (L1 and L1 regularized), decision tree, random forest, AdaBoost, gradient boosting machine (GBM), and lightGBM, to predict the class of intravenous immunoglobulin resistance (binary classification). Data from patients who were discharged before Sep 2018 were included in the training set (n = 497), while all the data collected after 9/1/2018 were included in the test set (n = 147). We used the area under the ROC curve, accuracy, sensitivity, and specificity to evaluate the performances of each model. The gradient GBM had the best performance (area under the ROC curve 0.7423, accuracy 0.8844, sensitivity 0.3043, specificity 0.9919). Additionally, the feature importance was evaluated with SHapley Additive exPlanation (SHAP) values, and the clinical utility was assessed with decision curve analysis. We also compared our model with the Kobayashi score, Egami score, Formosa score and Kawamura score. Our machine learning model outperformed all of the aforementioned four scoring models. Our study demonstrates a novel and robust machine learning method to predict intravenous immunoglobulin resistance in Kawasaki disease patients. We believe this approach could be implemented in an electronic health record system as a form of clinical decision support in the near future.

## Introduction

Kawasaki disease (KD) is a self-limited systemic vasculitis that predominantly affects children under 5 years old. Tomisaku Kawasaki first reported on KD in 1967. Fifty years after the first report of KD, the cause of the disease remains unknown. The incidence of KD varies from 3.4 to 218.6 cases per 100,000 children. The incidence in certain Asian countries (Japan, Korean, China) is significantly higher than that in Western countries. The incidence worldwide has exhibited an increasing trend in the last few decades [1]. Clinical features of KD include persistent fever, cervical lymphadenopathy and mucocutaneous changes. Most clinical features resolve in 4 weeks even without treatment. However, KD is still the leading cause of pediatric acquired heart disease because of its main complication, coronary artery abnormalities (CAAs). CAAs also contribute the most to the mortality of KD patients [1].

Currently, the most effective therapy is high doses (2 g/kg) of intravenous immunoglobulin (IVIG) and aspirin. Timely initiation of the treatment can reduce the incidence of coronary artery aneurysms from 25% to approximately 4%. However, approximately 10% to 20% of treated children have persistent or recurrent fever after the first infusion of IVIG, indicating IVIG resistance. Many studies have shown that IVIG-resistant patients are at a greater risk of developing CAAs [1–5]. Intravenous methylprednisolone (IVMP) is an important rescue treatment after patients fail to respond to initial therapy for KD. IVMP treatment in the acute phase of KD reduces the incidence of CAAs [6, 7]. A recent Cochrane review [8] suggests that giving a patient IVMP during the initial treatment will have more favorable effects than giving it during the rescue treatment period. Other rescue treatments, such as infliximab, also have different outcomes when used during the initial treatment period [9, 10]. Considering the possibility that glucocorticoids might worsen coronary artery disease [11] and the high cost of infliximab, IVMP and infliximab cannot be recommended routinely as a component of initial therapy to all KD patients. Thus, the prediction of IVIG resistance would allow the use of additional therapies in the early stage and make the prevention of CAAs possible.

Several scoring models have been established to predict resistance to IVIG. The Kobayashi score [12], a well-known model published in 2006, is a logistics regression scoring model. The Kobayashi score comprises seven variables, including serum sodium (Na) ≤133 mmol/L, days of illness at the initial treatment≤4, aspartate aminotransferase (AST) ≥100 IU/L, N%80%, c-reactive protein (CRP) ≥10 mg/dL, age≤12 months and platelet count (PLT) ≤30*104/mm3. In the same year, Egami and his colleagues generated another predictive scoring model [13], which includes 5 variables. The Egami and Kobayashi scores share 4 of the same variables. Many study groups have evaluated these two well-known scoring models in different regions outside of Japan. Both scoring models have an acceptable validation outcome in East China [14] but showed a relatively unsatisfactory performance in North America [15], North China [16, 17], Israel [18] and Italy [19]. Thus, studies on IVIG resistance prediction have accumulated in recent years in different regions. The Formosa score, based on the Taiwan population, was developed in 2015 [20]. In 2016, Kawamura developed another scoring model [21]. However, until now, none of those models have been sufficiently accurate enough to be widely clinically useful in predicting the response of IVIG in the initial treatment. Developing a better predictive model for different regions is still a challenge.

Machine learning algorithms have been developed in recent decades. The learning tasks can be summarized in two categories: (1) regression, i.e., estimating a new value according to existing values, and (2) classification, i.e., predicting the outcomes according to the existing covariates. The learning algorithms can be linear or nonlinear and could be a single model or an ensemble learning model. Machine learning algorithms have been applied to many different fields and have shown great potential in assisting clinical diagnosis. Here, we propose a machine learning approach to find a better model to predict IVIG resistance in KD patients.

## Materials and methods

### Data description

To obtain the data and build the prediction model, we retrospectively collected the medical records of KD patients hospitalized in the Fujian Provincial Maternity and Children’s Hospital from the electronic health record system from March 2013 to June 2019. Fujian Province is a southeast coastal province of China. Fujian Provincial Maternity and Children’s Hospital is a tertiary specialized hospital that serves the majority of children in this area, which contains approximately 40 million people. The study was approved by the Ethics Committee of Fujian Provincial Maternity and Children’s Hospital (No: 2019-165). All data were fully anonymized before we accessed them. The ethics committee waived the requirement for informed consent.

The diagnosis of KD was based on the American Heart Association (AHA) guideline criteria [1, 22]. Complete KD is confirmed when patients have fever for more than 5 days plus at least 4 of 5 of the following principal clinical features: 1. Rash, 2. Bilateral conjunctive injection, 3. Cervical lymphadenopathy, 4. Changes in the extremities, and 5. Oral mucosal changes. We diagnosed incomplete KD when patients had ≥5 days of fever and 2 or 3 compatible clinical criteria and had CRP≥30 mmol/L or ESR ≥40 mm/h; then, we evaluated CAAs based on echocardiography or a set of suspicious laboratory criteria according to the guidelines. IVIG resistance is defined as recrudescent or persistent fever ≥36 h after the end of the IVIG infusion.

All patients in the dataset received 2 g/kg IVIG in one day or 1 g/kg IVIG separated between two days. The difference in the usage of IVIG was documented. Aspirin was given at 30-50 mg/kg as recommended by the guidelines, and the dose was then decreased to 3–5 mg/kg/day when patients were afebrile for 48 to 72 h.

**Fig 1:**
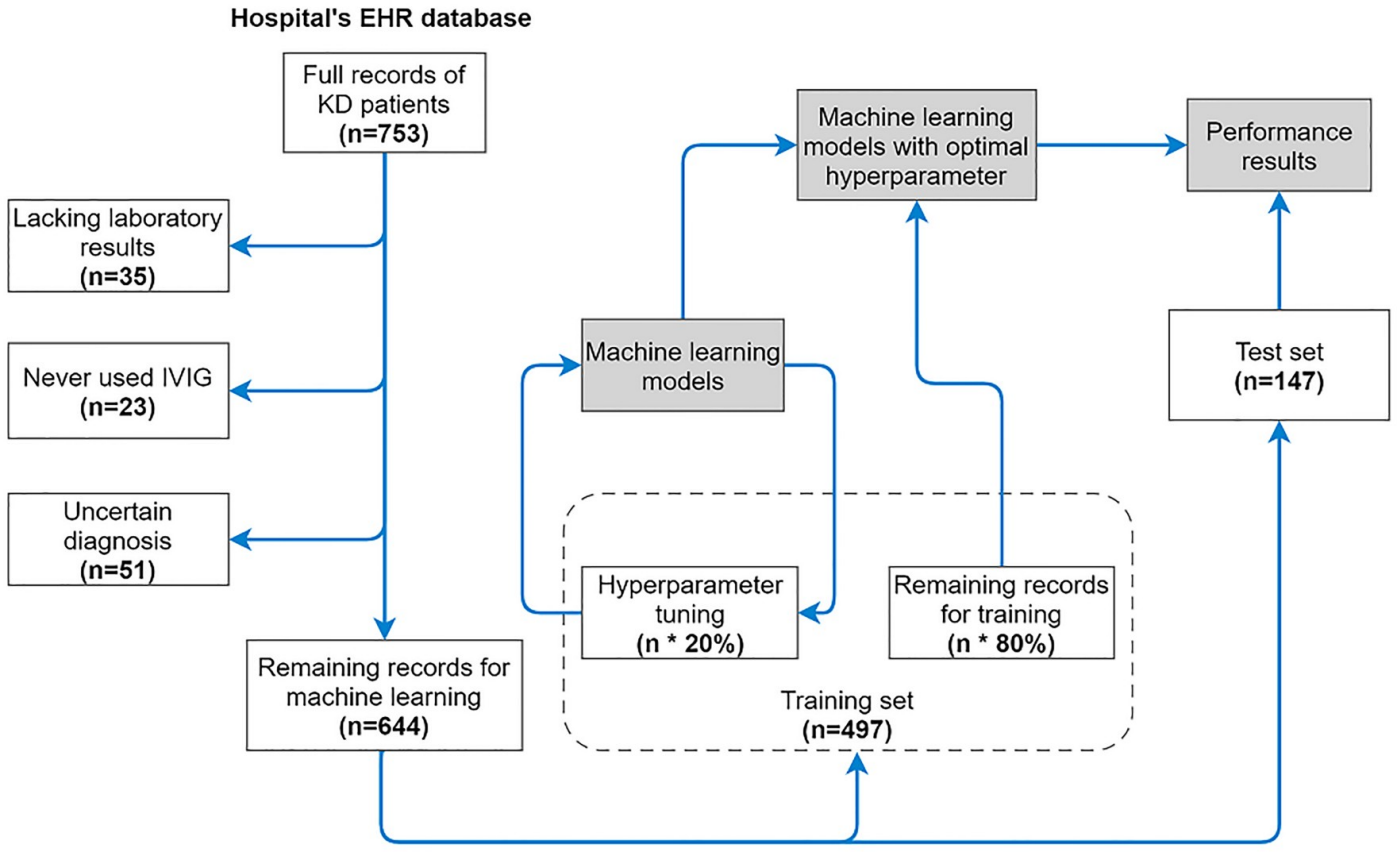
Schematic of patient enrollment and development of the machine learning model. A full record contains 82 features. There were 644 remaining records after the data cleaning process. The sizes of the training set and test set are 497 and 147, respectively. The hyperparameter tuning process uses 20% records in the training set. Machine learning models with optimal hyperparameters used the other 80% of records for training. Records in the test set (n = 147) were used to test the results of the trained models.

We collected 753 observations in total. Thirty-five patients were excluded from the dataset due to a severe lack of laboratory results, and 23 patients were excluded because they were never treated with IVIG during their hospitalization. Fifty-one patients were excluded because of disagreement with the AHA guidelines (some of them were diagnosed by Japanese guideline criteria). The remaining 644 observations (Demographic and clinical features in Table 1. Full recod see S1 Dataset on GitHub) form the dataset; 124 of the patients were IVIG resistant (19.25%). Data from patients discharged before Sep 2018 were considered the training set (n = 497), while all the data collected after Sep 2018 were considered the testing set (n = 147, IVIG resistance = 15.65%) (Fig 1).

**Table 1:** Demographic and clinical features of patients.

Categories	Variables	Data
**Age (mean ± SD)**	Age in months	19.4 ± 16.8
**Sex (n, %)**	Male	400 (62.1%)
	Female	244 (37.9%)
**Season (n, %)**	Spring	128 (19.9%)
	Summer	234 (36.3%)
	Autumn	151 (23.4%)
	Winter	151 (23.4%)
**Clinical features (n, %)**	Rash	526 (81.7%)
	Erythema of oral mucosa	534 (82.9%)
	Strawberry tongue	430 (66.8%)
	Cervical lymphadenopathy	373 (57.9%)
	Edema of the hands and feet	312 (48.4%)
	Periungual desquamation	78 (12.1%)
	Total days with fever (mean ± SD)	7.4 ± 3.0
**Presentation (n, %)**	Typical	374 (58.1%)
	Atypical	270 (41.9%)
**IVIG responsiveness (n, %)**	Respond	520 (80.8%)
	Resistant	124 (19.2%)

Abbreviations: SD stands for Standard Deviation.

### Variables considered in the model

From the medical records of KD patients, we composed the dataset with four dimensions: basic information, clinical features, sonography measurement and laboratory results.

The basic information consisted of sex, age at diagnosis, body weight and body surface area.

Clinical features include days of illness prior to hospitalization, days of illness at diagnosis, sampling time, KD type, IVIG response, days of illness at the initial treatment, usage of IVIG, usage of glucocorticoids, changes around the anus, and all principal clinical features mentioned in the guidelines.

All sonography measurements were performed by pediatric radiologists with IE33 and IE Elite machines (PHILIPS, Amsterdam, Netherlands). Ultrasound examination reports were supervised by experienced consultant pediatric radiologists with over 10 years of sonographic experience. Coronary arterial diameter and coronary arterial Z score were measured under the recommendation and standard of The Child Coronary Arterial Diameter Reference Study Group of the Japan Kawasaki Disease Society [23]. The AHA Z score was also calculated and included [1].

Laboratory results include complete blood count, comprehensive metabolic panel, CRP and erythrocyte sedimentation rate (ESR). Complete blood count and CRP were obtained from K2-EDTA tubes using automated hematology analyzers (XS-1000i and XN-3000, Sysmex, Kobe, Japan). A comprehensive metabolic panel was performed using ARCHITECT ci16200 (Abbott Laboratories, Chicago, America). ESR was measured by CD3700 (Abbott Laboratories, Chicago, America). Specific ratios, such as neutrophil-to-lymphocyte and platelet-to-lymphocyte ratios, were calculated afterward.

In total, 82 features were collected (Table 2). In the procedure of building predictive models, machine learning algorithms allowed us to find unknown complicated relationships between these features and IVIG resistance in KD patients.

**Table 2:** Variables considered in the model.

Categories	Variables
**Basic information**	Sex, Age in months^1^^,^^2^, Weight, Body surface area
**Clinical features**	Days of illness prior to hospitalization, Days of illness at diagnosis, KD type, Rash, Erythema of oral mucosa, Strawberry tongue, Conjunctival injection, Cervical lymphadenopathy^4^, Edema of the hands and feet, Periungual desquamation, Changes around the anus, Days of illness at the initial treatment^1^^,^^2^, Usage of IVIG, Usage of glucocorticoids, Sampling stage
**Sonography measurement (acute stage)**	Left main coronary artery diameter, Proximal right coronary artery (RCA) diameter, Left main coronary artery Z_Score (Japan), Proximal_RCA Z_Score (Japan), Left main coronary artery Z_Score (AHA), Proximal_RCA Z_Score (AHA)
**Comprehensive metabolic panel**	Serum potassium, triglyceride, Blood urea nitrogen, Creatinine, Serum calcium, Alkaline phosphatase, Serum total protein, Serum albumin^4^, Serum globulin, Serum sodium^1^, Alanine aminotransferase^2^, Aspartate aminotransferase^1^, Gamma glutamyl transferase, Total bilirubin, Direct bilirubin, Indirect bilirubin, Serum magnesium, Lactic acid dehydrogenase, Creatine kinase, Creatine kinase-MB, Serum phosphorus, Cholesterol, Serum chlorine, Serum glucose, Carbon dioxide combining power, Erythrocyte sedimentation rate
**Complete blood count**	White blood cell count, Eosinophilic granulocyte count, Basophil count, Erythrocyte mean corpuscular volume, Mean corpuscular hemoglobin, Mean corpuscular hemoglobin concentration, Mean platelet volume, Plateletcrit, Percentage of monocytes, Percentage of eosinophils, Percentage of basophils, Neutrophil count, Hematocrit, Platelet distribution width, Percentage of lymphocyte, Percentage of neutrophils^1^^,^^4^, Red blood cell count, Platelet count^1^^,^^2^, Hemoglobin, Lymphocyte count, Monocyte count, Platelet larger cell ratio, Neutrophil-to-lymphocyte ratio^3^, Platelet-to-lymphocyte ratio^3^
**Others**	C-reactive protein^1^^,^^2^, Erythrocyte sedimentation rate

^1^: Variables used in Kobayashi score.

^2^: Variables used in Egami score.

^3^: Variables used in Kawamura score.

^4^: Variables used in Formosa score.

### Machine learning-based classification algorithms

Two linear models (logistic regression with L1 and L2 regularization) were used as baseline algorithms, and the remaining 5 models (decision tree, random forest, AdaBoost, GBM, and lightGBM) were used to compare the baseline algorithms (see code S1 Code). In this work, we are particularly interested in the gradient boosting machine algorithm as it achieves the highest accuracy.

To evaluate the performances of each model, we used 4 different metrics: Area under the ROC curve (AUC), accuracy, sensitivity, and specificity to test on the validation or testing sets. Basically, AUC is equal to the probability that a classifier will rank a randomly chosen positive instance higher than a randomly chosen negative one. Accuracy is measured by the percentile of correct classified observations over all observations. Sensitivity is the ability of a learner to correctly identify the positive target, whereas the specificity is the ability of a learner to correctly identify the negative target. All four metrics ranges from 0 to 1, the higher the better.

All machine learning models were assigned imbalanced weight according to the proportion of positive results in the training dataset [24, 25] except GBM since GBM didn’t implemented the model imbalance technique. Specifically, we gave 0.2 and 0.8 values to negative and positive weights, respectively.

### Gradient boosting machine

Gradient boosting machine (or classifier, GBM in short) is a machine learning algorithm for regression and classification tasks. It ensembles multiple weak predictors, which are typically decision trees, to form a strong predictor [26]. We adopted Scikit-learn package [27] in python 3.7 environment to implement the gradient boosting machine.

### Model explanation

Taking an example of the GBM, the model was driven by the loss of deviance. Specifically, for 2-class classification, binomial deviance was used. Let P be the log odds; then, the definition of the binomial deviance of an observation is

$$\begin{array}{c} ylog\left(p\right)+\left(1-y\right)log\left(1-p\right)=log\left(1-p\right)+ylog\left(\frac{p}{1-p}\right) \end{array}$$ (1)

Now observe that $p=\frac{e^{P}}{1+e^{P}}$ and $1-p=\frac{1}{1+e^{P}}$, thus

$$\begin{array}{c} log\left(1-p\right)=log\left(\frac{1}{1+e^{P}}\right)=-log\left(1+e^{P}\right) \end{array}$$ (2)

and

$$\begin{array}{c} log\left(\frac{p}{1-p}\right)=log\left(e^{P}\right)=P \end{array}$$ (3)

Thus, the binomial deviance is equal to

$$\begin{array}{c} y^{P}-log\left(1+e^{P}\right) \end{array}$$ (4)

where *P* stands for log odds of predicted labels, *p* stands for the probability of an event occurring, and *y* stands for true labels. Binomial deviance loss is more robust in noise-prone situations and thus was adopted in the GBM optimization.

### Prameter setting

In the GBM algorithm, the loss function was set to deviance, the learning rate was set to 0.1, and the criterion was set to Friedman’s mean squared error (MSE). The minimum number of samples required to split an internal node was set to 2, and the minimum number of samples required to be at a leaf node was set to 1. The maximum depth of each individual regression estimator was set to 3. The maximum depth limits the number of nodes in the tree. A deeper tree might generalize a better performed result but may also lead to overfitting. The minimum impurity split was set to 1 × 10^−7^. The number of estimators in this experiment was tuned during the hyperparameter tuning section. The number of estimators can be 16, 32, 64, or 128.

### Hyperparameters tuning

Hyperparameters were tuned according to the validation set. In the training set, we randomly picked 20% of the data for the validation set for tuning the hyperparameters. In logistic regression with L1 and L2 regularization, the hyperparameter is the regularization strength, and we searched 0.5, 1, 1.5, 2, 2.5, and 3. In the decision tree, the hyperparameter is the depth of the tree. We searched from depth = 5 to depth = 15. In ensemble learning methods, including random forest, AdaBoost, and the GBM, the hyperparameter is the number of ensemble estimators. We searched 16, 32, 64, and 128. In lightGBM, we searched the depth of the trees from depth = 5 to depth = 15. The optimal hyperparameters were determined according to the best AUC in the validation set. Once the optimal hyperparameters were determined, we combined the training and validation sets together and reran the learning algorithm. Finally, the performances of the above models were evaluated on the holdout testing set using tuned hyperparameters.

### Results

**Fig 2:**
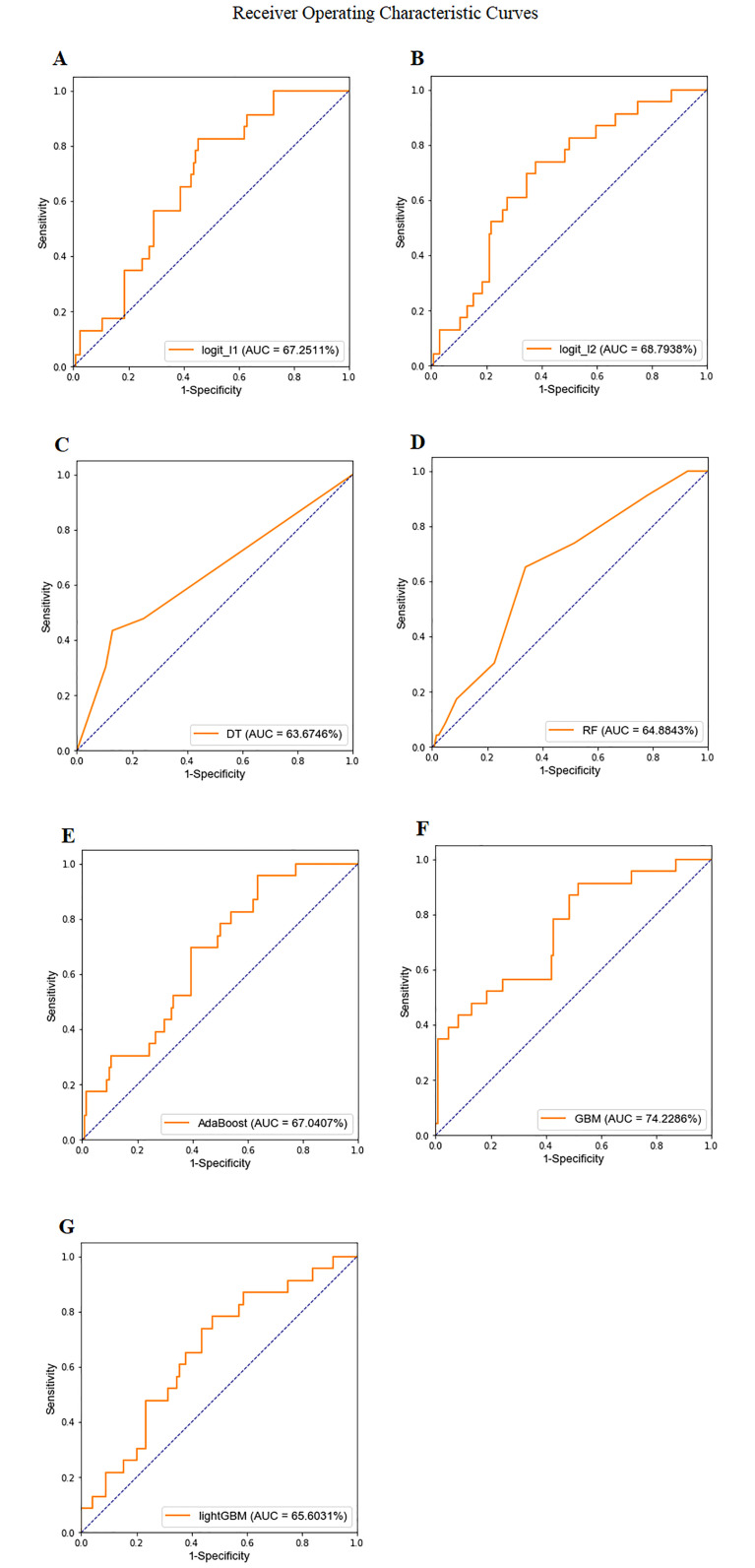
ROC curves and AUC values: An analysis of the predictive capacity for discrimination between IVIG-resistant and non-IVIG-resistant KD patients. A: Logistic regression with L1 regularized (AUC 0.6725). B: Logistic regression with L2 regularized (AUC 0.6879). C: decision tree with maximum depth = 12 (AUC 0.6367). D: random forest with 16 estimators (AUC 0.6488). E: AdaBoost with 64 estimators (AUC 0.6704). F: GBM with 32 estimators (AUC 0.7423). G: lightGBM with maximum depth = 5 (AUC 0.6560).

Fig 2 shows the ROC curves and AUC results. We found that the ensemble-based GBM algorithm achieved the optimal AUC (0.7423) on the testing set, suggesting that the ensemble-based GBM algorithm performed the best.

In Table 3, we further examine the detailed performances of 7 machine learning algorithms according to 4 evaluation metrics. We found that the highest AUC and accuracy were achieved by GBM; however, the highest sensitivity was achieved by the decision tree. The best hyperparameters for each model are given at the bottom.

**Table 3:** Model performances in AUC, accuracy, sensitivity, and specificity.

model	logit_l1	logit_l2	DT	RF	AdaBoost	GBM	lightGBM
**AUC**	0.6725	0.6879	0.6367	0.6488	0.6704	**0.7423**	0.6560
**accuracy**	0.7007	0.7415	0.7143	0.8367	0.7959	**0.8844**	0.7619
**sensitivity**	0.3478	0.2609	**0.4783**	0.0435	0.3044	0.3043	0.2174
**specificity**	0.7661	0.8306	0.7581	0.9839	0.8871	**0.9919**	0.8629
**best hyper-parameter**	1	3	12	16	64	32	5

Abbreviations: Logit l1 and logit l2 represent logistic regression with L1 and L2 regularizations, respectively; DT stands for decision tree; RF stands for random forest; GBM stands for gradient boosting machine.

We performed chi-square tests between the GBM model and each of the other 6 machine learning models. The purpose of the Chi-square test is to calculate the probability (P) of the observed outcomes would occur when assuming there is no difference in performance of models. The results show that the GBM model is significantly different from the L1 regularization logistic regression, L2 regularization logistic regression, decision tree, Adaboost, and lightGBM models (P<0.01). No statistical significance was found between the random forest and GBM models. Because of the extremely low sensitivity (0.0435) of the random forest model, more observations may be needed to detect a significant difference.

Finally, we conclude that GBM performed better than the baseline logistic regressions, suggesting nonlinear interactions among the input covariates (features).

### Clinical utility assessment

We used decision curve analysis [28] to assess the clinical utility of machine learning models. A decision curve is generated from the net benefit plotted against a range of threshold probabilities. The threshold probability is a level of certainty above which the patient or physician would choose to intervene, which is a subjective variable. The threshold probability is lower when the patient or physician is more concerned about the disease, while it is higher when the patient or physician is more concerned about interventions. The net benefit is calculated as

$$\begin{array}{c} Net\;benefit=\frac{True\;positive}{N}-\frac{False\;positive}{N}\times \frac{P_{t}}{1-P_{t}} \end{array}$$ (5)

where *N* represents the total sample size and *P*_*t*_ represents the threshold probability [29].

Decision curves include benefits and harms on the same scale so that they can be compared directly, supporting the clinical choice between models.

**Fig 3:**
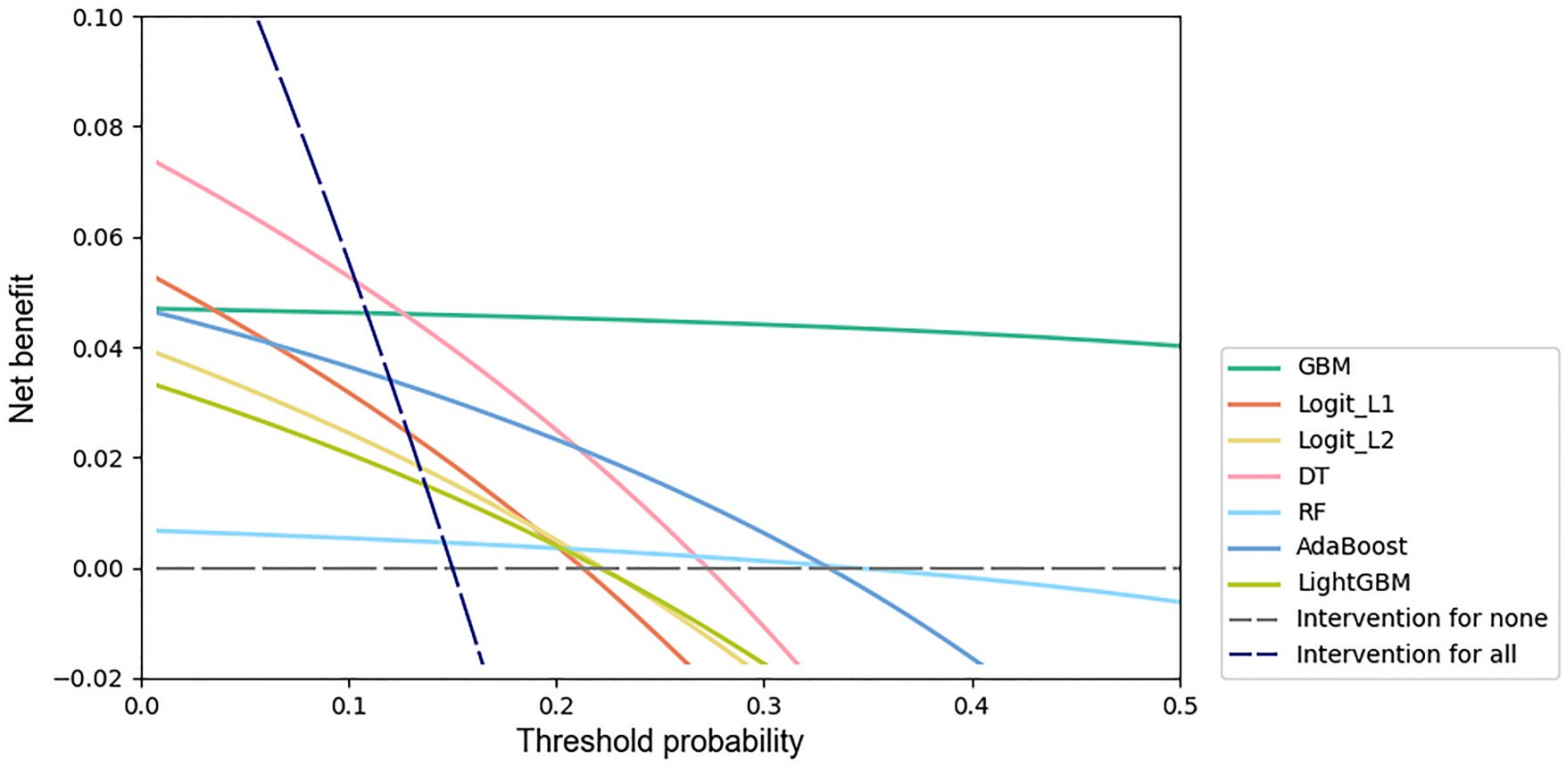
Decision curves for predicting IVIG resistance in KD patients by machine learning models. The x-axis indicates the threshold probability for the outcome of IVIG resistance among KD patients without additional initial treatment. The y-axis indicates the net benefit. Two extreme strategies, intervention for all and intervention for none, were added as references.

We performed decision curve analyses of all machine learning models, and the results are shown in (Fig 3). The net benefit for the GBM model was greatest across the range of threshold probabilities higher than 13% (12.66%) compared with the net benefit for the other machine learning models.

### Feature importance

**Fig 4:**
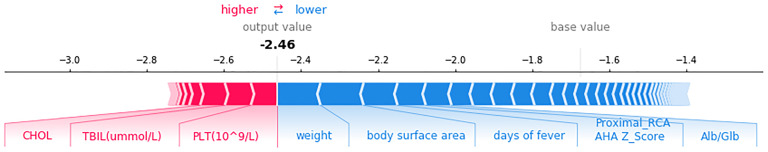
SHAP force plot. Features contributing to pushing the model toward the output from the base value (the average model output over the training dataset we passed). Features pushing the prediction higher are shown in red, and those pushing the prediction lower are shown in blue.

**Fig 5:**
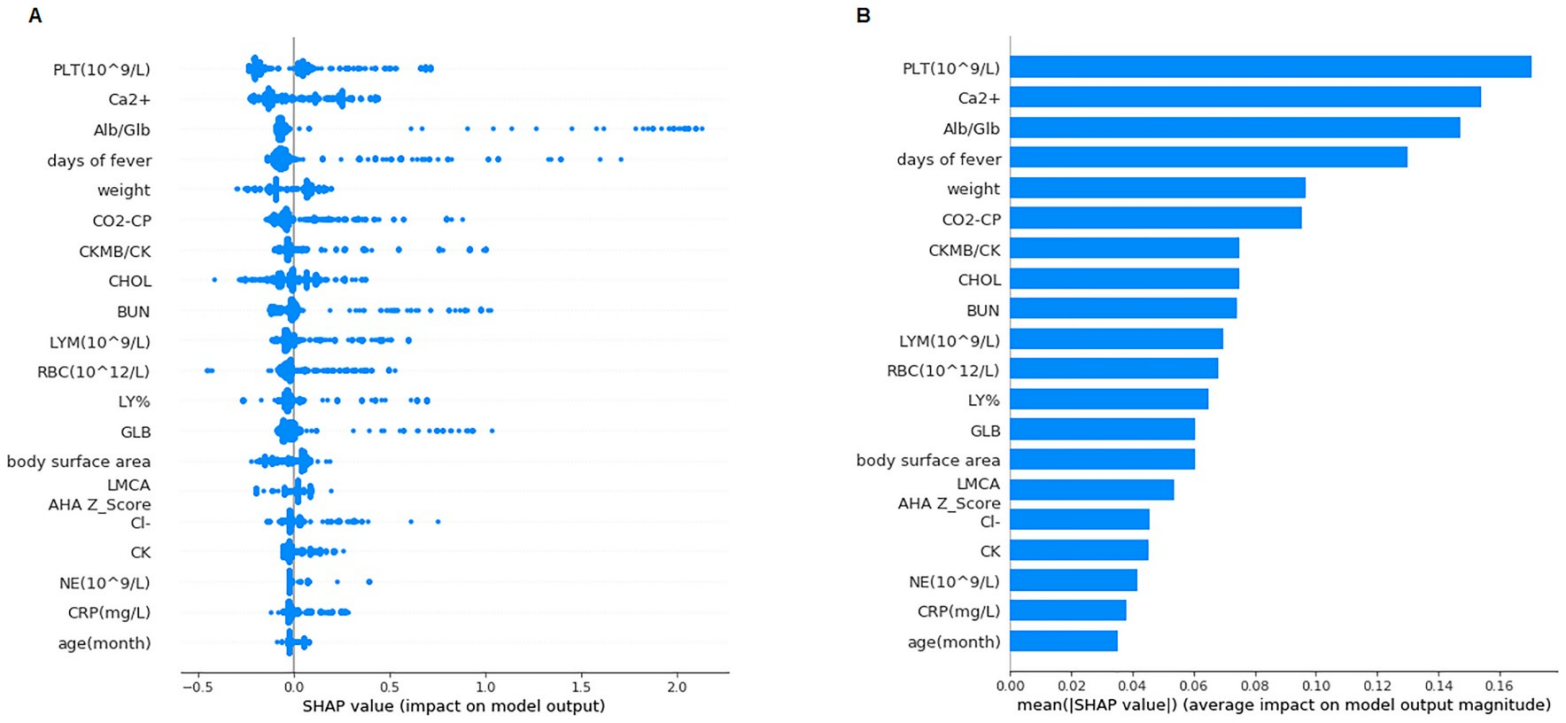
SHAP values and importance. A: The SHAP values of the 20 most important features for every sample. Features are sorted in descending order by Shapley values. B: Feature importance represented by the mean absolute Shapley value. Features are sorted in descending order by Shapley values.

Next, we used SHapley Additive exPlanation (SHAP) to quantify and rank the importance of features, leveraging the idea of Shapley values for model feature influence scoring. SHAP values consistently consider feature importance, better align with human intuition, and better recover influential features [30]. A higher value indicates that a feature has a larger impact on the model, which makes this feature more important. According to the GBM model, the SHAP force is presented in (Fig 4), and the most important features are presented in Fig 5A and 5B.

We found that the top features were platelet count, blood calcium, albumin-to-globulin ratio, days of fever prior to hospitalization, and body weight. The three highest Shapley value features that pushed the prediction higher are platelet count, total bilirubin and cholesterol. These features play pivotal roles in helping the machine learning algorithm construct decisions. To verify the feature importance, we excluded the top 15 features in Fig 5A from the dataset and reprocessed GBM model training and testing. The output model had a relatively poor performance (AUC 0.6413), emphasizing the importance of these features.

### Assessment of scoring models

We also compared the Kobayashi score, Egami score, Formosa score and Kawamura score with our test set. Variables that are considered in aforementioned models are showed in Table 2. The results are presented in Table 4. The GBM model we trained is listed for comparison. Chi-square tests were performed and showed significant differences between our GBM model and each of those four scoring models (P<0.01), indicating that the performance differences are statistically significant.

**Table 4:** Model performances comparison.

model	accuracy	sensitivity	specificity	PPV	NPV	AUC
**Kobayashi**	0.5782	0.1304	0.6613	0.0667	0.8039	0.5700
**Egami**	0.6735	0.1304	0.7742	0.0968	0.8276	0.6520
**Formosa**	0.7211	0.4348	0.7742	0.2632	0.8807	0.5070
**Kawamura**	0.5646	0.4782	0.5806	0.1746	0.8571	0.5050
**Ours**	0.8844	0.3043	0.9919	0.8750	0.8848	0.7423

Performance of Kobayashi score, Egami score, Formosa score and Kawamura score in accuracy, sensitivity, specificity, positive predictive value (PPV), negative predictive value (NPV) and AUC. The GBM model we trained is listed for comparison.

**Fig 6:**
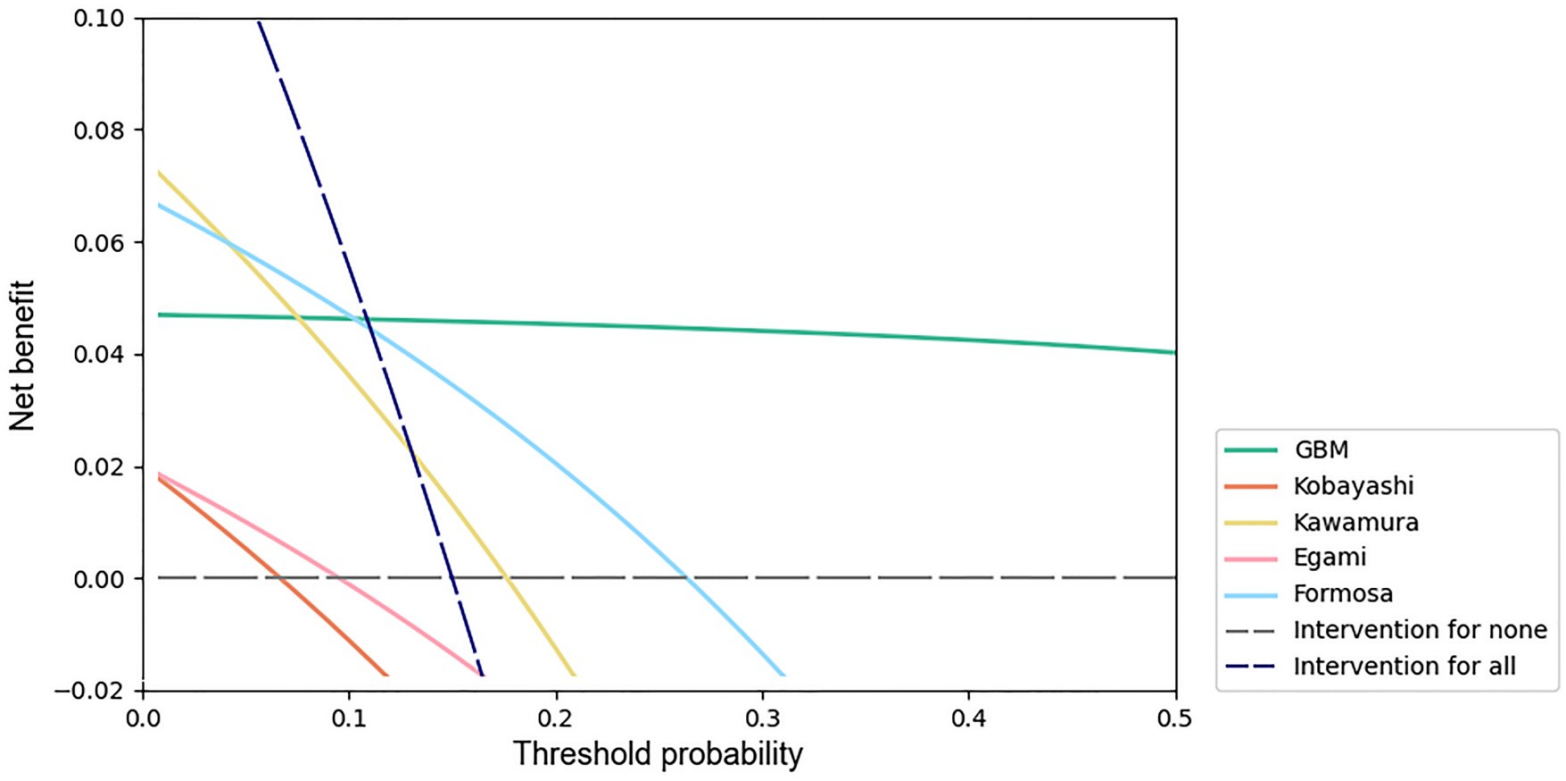
Decision curves of the GBM model, Kobayashi score, Egami score, Formosa score and Kawamura score.

We applied decision curve analysis to the Kobayashi score, Egami score, Formosa score and Kawamura score with our test set. The results are shown in Fig 6. The net benefit of the GBM model was greatest across the range of threshold probabilities higher than 11% (10.88%).

## Discussion

We used 7 different machine learning algorithms to classify the outcome of IVIG resistance. The results suggest that GBM achieves optimal performance in the measurement of AUC and accuracy in the testing set. The optimal hyperparameter was 32 base estimators and was determined according to the performance of the validation set. By importing a KD patient’s basic test results into our trained model, the clinician will be able to know whether the patient is IVIG resistant.

In the past decade, logistic regression has been the first choice to build IVIG prediction models. The Kobayashi score, Egami score, Formosa score and most other predictive scores were based on logistic regression. With the rapid development of machine learning algorithms and model-explaining methods, there are many efficient algorithms to choose now. For the first time, multiple machine learning algorithms were compared in research on KD. We offered an insightful approach to researchers in this field. The code can be found on our GitHub page.

Our Python code was initially run under the Linux environment. However, when we tested it under the Microsoft Windows 10 environment, lightGBM achieved a different performance (AUC 0.7812, accuracy 0.8708, sensitivity 0.2609, specificity 0.9838), while the performance of the other 6 models remained the same. One reason could be that the source codes of lightGBM in different operating systems are different. Due to the unstable performance of lightGBM, we still concluded that GBM is a better-performing algorithm.

Decision curve analyses showed that the net benefit for the GBM model was greatest across the range of threshold probabilities higher than 13%. Adverse effects of IVMP therapy in patients with KD include infection, gastrointestinal hemorrhage, hypercoagulability, sinus bradycardia, hypertension, hyperglycemia, hypothermia and secondary adrenocortical insufficiency. Considering all adverse effects of IVMP and the possibility that it might worsen coronary artery disease, it is reasonable to set the threshold probability of initial IVMP therapy at approximately 30%. Using the GBM model at this threshold probability is a better option. Since threshold probability is a subjective variable, it could be reset by patients and physicians together according to specific conditions.

Based on the SHAP value derived from GBM, we found that features such as platelet count, blood calcium, albumin-to-globulin ratio, days of fever prior to hospitalization, body weight, total bilirubin and cholesterol played pivotal roles in the learning process, while other features did not have a strong ability to help the classifiers.

Platelet count was reported as a risk factor for IVIG resistance and CAAs in KD patients by Dr. Kobayashi and Dr. Egami in a previous study. A theory is that coronary vascular endothelium damage may result in the activation of platelets, which establishes a cascade of further vascular damage [1], which may be reflected in platelet count. Albumin and globulin are the two major proteins found in the blood, and while albumin is primarily produced in the liver, globulin can also be produced by the immune system. An abnormal albumin-to-globulin ratio is connected with autoimmune diseases and liver problems. Hepatobiliary dysfunction and its association with IVIG resistance in KD patients have been described previously [31, 32]. The mechanism remains unclear and could be due to generalized inflammation, vasculitis of small and medium-sized vessels, toxin-mediated effects, or a combination of the above [31]. Blood calcium changes in KD patients have not been discussed frequently before. Andrew M. Kahn and his colleagues reported that coronary artery calcium scoring had a good performance in detecting coronary artery abnormalities in KD patients [33], but the relationship between coronary artery calcium scoring and blood calcium is still uncertain [34, 35].

In summary, we performed an analysis of data to predict the outcome of IVIG resistance in KD patients by implementing 7 different machine learning algorithms. Hyperparameters were tuned according to the validation set. We found that GBM is the best performing algorithm. This work helped us to identify the best machine learning model to predict IVIG resistance and suggested the importance of the features. Our study demonstrates a novel strategy to predict IVIG resistance in KD patients using a machine learning approach. We believe this approach could be implemented in an electronic health record system as clinical decision support in the near future. Nevertheless, more data including additional features, such as genetic variants, may help us to improve our model.

## Supporting information

S1 Dataset All data used to train and test. (XLSX)

S1 Code Code to train and compare 7 different machine learning models. (PY)
