# Correction: A machine learning approach to predict intravenous immunoglobulin resistance in Kawasaki disease patients: A study based on a Southeast China population

**DOI:** 10.1371/journal.pone.0253675

**Published:** 2021-06-21

**Authors:** Tengyang Wang, Guanghua Liu, Hongye Lin

There is an error in affiliation 1 for authors Tengyang Wang and Guanghua Liu. The correct affiliation 1 is: Department of pediatrics, Fujian Provincial Maternity and Children’s Hospital, Affiliated Hospital of Fujian Medical University, Fuzhou, Fujian, China

## References

[1] Wang T, Liu G, Lin H (2020) A machine learning approach to predict intravenous immunoglobulin resistance in Kawasaki disease patients: A study based on a Southeast China population. PLoS ONE 15(8): e0237321. 10.1371/journal.pone.0237321 32853226 PMC7451628

